# Influence of Green, Red and Blue Light Emitting Diodes on Multiprotein Complex Proteins and Photosynthetic Activity under Different Light Intensities in Lettuce Leaves (*Lactuca sativa* L.)

**DOI:** 10.3390/ijms15034657

**Published:** 2014-03-17

**Authors:** Sowbiya Muneer, Eun Jeong Kim, Jeong Suk Park, Jeong Hyun Lee

**Affiliations:** Department of Horticulture, College of Agricultural Life Sciences, Chonnam National University, 300 Young Bong-Dong Buk-Gu, Gwangju, 500-757, Korea; E-Mails: sobiyakhan126@gmail.com (S.M.); ejldj@hanmail.net (E.J.K.); bjs5187@naver.com (J.S.P.)

**Keywords:** lettuce (*Lactuca sativa* L.), light emitting diodes, photosynthesis, stomata, BN-PAGE

## Abstract

The objective of this study was to investigate the response of light emitting diodes (LEDs) at different light intensities (70 and 80 for green LEDs, 88 and 238 for red LEDs and 80 and 238 μmol m^−2^ s^−1^ for blue LEDs) at three wavelengths in lettuce leaves. Lettuce leaves were exposed to (522 nm), red (639 nm) and blue (470 nm) LEDs of different light intensities. Thylakoid multiprotein complex proteins and photosynthetic metabolism were then investigated. Biomass and photosynthetic parameters increased with an increasing light intensity under blue LED illumination and decreased when illuminated with red and green LEDs with decreased light intensity. The expression of multiprotein complex proteins including PSII-core dimer and PSII-core monomer using blue LEDs illumination was higher at higher light intensity (238 μmol m^−2^ s^−1^) and was lowered with decreased light intensity (70–80 μmol m^−2^ s^−1^). The responses of chloroplast sub-compartment proteins, including those active in stomatal opening and closing, and leaf physiological responses at different light intensities, indicated induced growth enhancement upon illumination with blue LEDs. High intensity blue LEDs promote plant growth by controlling the integrity of chloroplast proteins that optimize photosynthetic performance in the natural environment.

## 1. Introduction

Plants use light as an energy source for photosynthesis and as an environmental signal, and respond to its intensity, wavelength, and direction. Light is perceived by plant photoreceptors that include phytochromes, cryptochromes and phototropins and plants generate a wide range of specific physiological responses through these receptors. A major challenge to plants is controlled by supplying sufficient quantity and quality of light intensities [1,2]. Light emitting diodes (LEDs) has been proposed as a light source for controlled environment agriculture facilities and space based plant growth chambers because they exhibit desirable characteristics such as small mass, safety and durability [3–5].

Plant development and physiology are strongly influenced by the light spectrum of the growth environment among which blue light is involved in a wide range of plant processes such as phototropism, photo-morphogenesis, stomatal opening, and leaf photosynthetic functioning [6]. Most studies assessing the effects of blue light (blue LEDs) on the leaf or whole plant have either compared the response to a broadband light source with response to blue deficient light [7] or compared plants grown under red light alone [5,8]. On the other hand, red LEDs emit a narrow spectrum of light (660 nm) that is close to the maximum absorbance for both chlorophyll and phytochromes. Although red light components have a great potential for use as a light source to drive photosynthesis, plants are adapted to utilize a wide-spectrum of light to control photosynthesis [9]. The green LEDs have reduced photosynthesis [10]. Several reports have assessed the efficiency and deficiency of green light on growth and development of plants. Frechilla *et al*. [11] demonstrated that a brief pulse of green light could oppose stomatal opening, while stomates open if green light is followed by blue light.

The absorption of blue and red light (LEDs) by plants has been measured as 90% [12] which indicates that plant development and physiology is strongly influenced by blue or red light [13]. In contrast, green light has been reported to be negative on physiological and developmental incomes [14]. Many studies have been reported on several crops grown under deficiency/efficiency or using a combination of red and blue light at different wavelengths [15,16] on growth and development of plants. Plants grown under blue light exhibit photosynthesis more similar to those grown under red light, such as chlorophyll *a* and *b* ratio [17,18], a greater site f content [18] and a greater ribulose-1,5-bisphosphate carboxylase/oxygenase (RuBisCO) content [19].

However, little is known on the integrity of combined effect of green, red and blue LEDs, with no experimental evidence available concerning the expression of multiprotein complexes for promotion of induction of photosynthesis. Presently, we grew lettuce plants (*Lactuca sativa* L.) under different light intensities at three wavelengths (given in Table 1) of green, red and blue LEDs and analyzed the expression of thylakoid multiprotein complex proteins (MCPs), opening and closing of stomata and major photosynthetic parameters. Photosynthetic-mediated proteins in sub-compartments of chloroplasts including stomatal opening and closing and photosynthetic activity responded most to blue LEDs of high light intensity. The response of photosynthesis was more sensitive in blue LEDs than red and green LEDs.

## 2. Results

### 2.1. Growth Analysis and Leaf Water Potential

**Figure 1.**
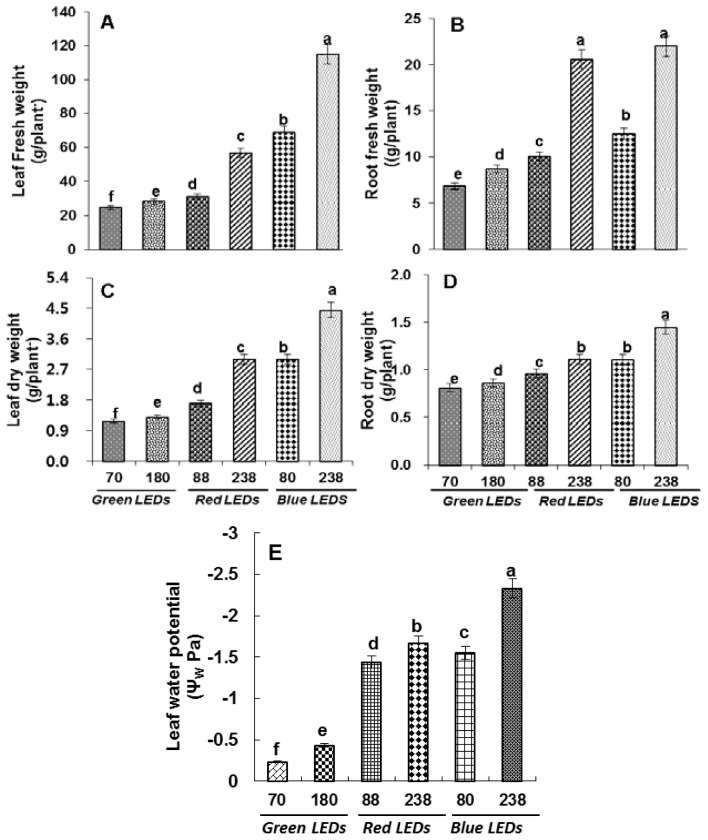
Growth parameters (**A**) Leaf fresh weight; (**B**) Root fresh weight; (**C**) Leaf dry weight; (**D**) Root dry weight and (**E**) Leaf water potential as affected by green, red and blue LEDs at different light intensities—green (70 and 180 μmol m^−1^ s^−1^), red (88 and 238 μmol m^−1^ s^−1^) and blue (80 and 238 μmol m^−1^ s^−1^). Vertical bars indicate ± SE of the means for *n* = 5. Means denoted by the different letter are significantly different at *p* < 0.05 according to the Tukey’s studentized range test.

We analyzed fresh and dry biomass of roots and leaves and it was observed that the biomass of plants grown under blue LEDs at high light intensity (238 μmol m^−2^ s^−1^) was significantly higher than low light intensity (80 μmol m^−2^ s^−1^). The biomass was observed to be low in plants grown under red and lowest under green LEDs with a decrease in light intensity (Figure 1A–D).

Ψ_W_ (water potential) reached a maximium of −2.3 Mpa in plants grown under blue LEDs at 238 μmol m^−2^ s^−1^ (Figure 1E) and a minimum of −0.23 MPa in leaves of plants grown under green LEDs at 91 μmol m^−2^ s^−1^.

### 2.2. Photosynthetic Activity, Stomatal Conductance, Fv/Fm Ratio, and Transpiration

**Figure 2.**
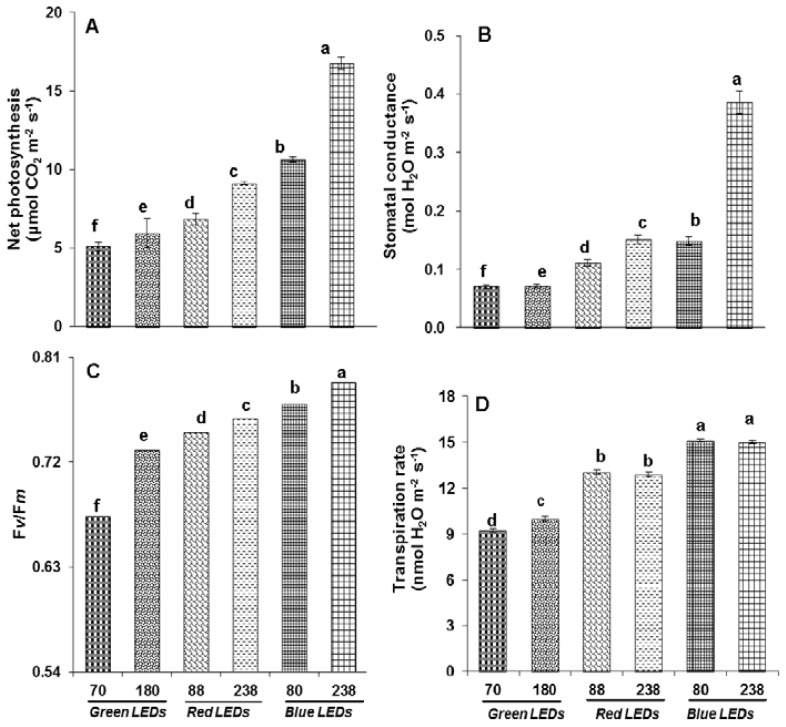
Changes in photosynthetic parameters (**A**) Net photosynthesis; (**B**) Stomatal conductance; (**C**) Fv/Fm; and (**D**) Transpiration rate as affected by green, red and blue LEDs at different light intensities—green (70 and 180 μmol m^−1^ s^−1^), red (88 and 238 μmol m^−1^ s^−1^) and blue (80 and 238 μmol m^−1^ s^−1^). Vertical bars indicate ± SE of the means for *n* = 5. Means denoted by the different letter are significantly different at *p* < 0.05 according to the Tukey’s studentized range test.

The plants grown at 238 μmol m^−2^ s^−1^ showed a significantly higher rate of photosynthesis (Figure 2A) than plants grown at 80 μmol m^−2^ s^−1^ under blue LEDs. However, the plants grown under red LEDs showed lower rates of photosynthesis with a decrease in light intensity. The lowest rate of photosynthesis was observed for the plants grown under green LEDs with a decrease in light intensity.

The observations of plants grown under blue LEDs at 238 μmol m^−2^ s^−1^ positively showed that induction of stomatal conductance, Fv/Fm and transpiration rate (Figure 2B–D) occurred moreso than for the plants grown under red LEDs. Under green LEDs, the stomatal conductance, Fv/Fm and transpiration rate was reduced compared to red and blue LEDs.

### 2.3. Stomatal Observations

**Figure 3.**
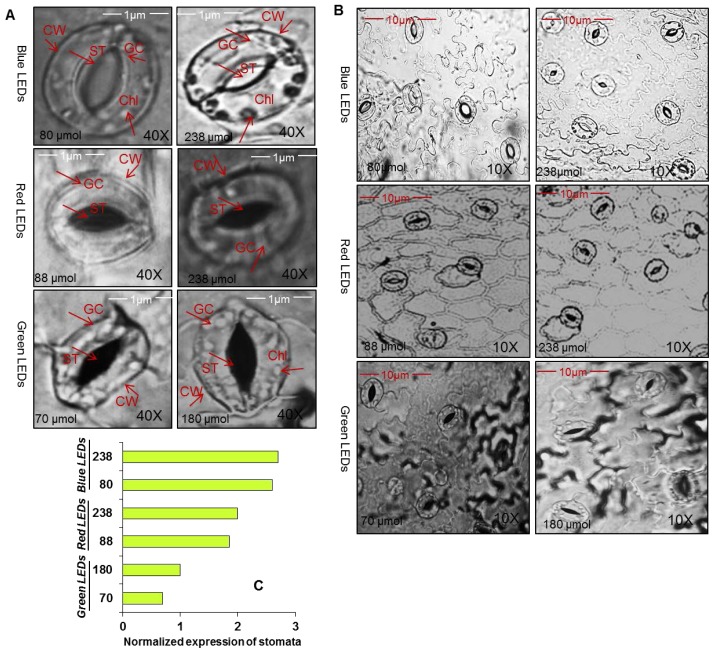
(**A**,**B**) Representative images; and (**C**) Normalized expression of stomata as affected by green, red and blue LEDs at different light intensities green (70 and 180 μmol m^−1^ s^−1^), red (88 and 238 μmol m^−1^ s^−1^) and blue (80 and 238 μmol m^−1^ s^−1^). Thin layer of leaf outer covering were peeled off carefully and laid on a glass slide, covered with a cover slip and were observed under a light microscope (Leica CME) at 40× magnification. CW indicates cell wall, ST indicates stoma, GC indicates guard cells, and Chl indicates chloroplast.

We observed stomata at different LEDs and different light intensities (Figure 3A–C). The plants grown under blue LEDs at 80 and 238 μmol m^−2^ s^−1^ showed well organized guard cells with open stomata and the number of stomata was also observed to be higher (see normalized expression). However, although the plants grown under green and red LEDs at different light intensities showed well organized guard cells, the stomata was observed to be closed and a reduction in the number of stomata was also observed. We also detected stomatal density (Table 1), and observed that under blue LEDs at 80 and 238 μmol m^−2^ s^−1^, stomatal density was higher compared to green and red LEDs at their respcteive light intensities.

### 2.4. Thylakoid Membrane Proteins

**Figure 4.**
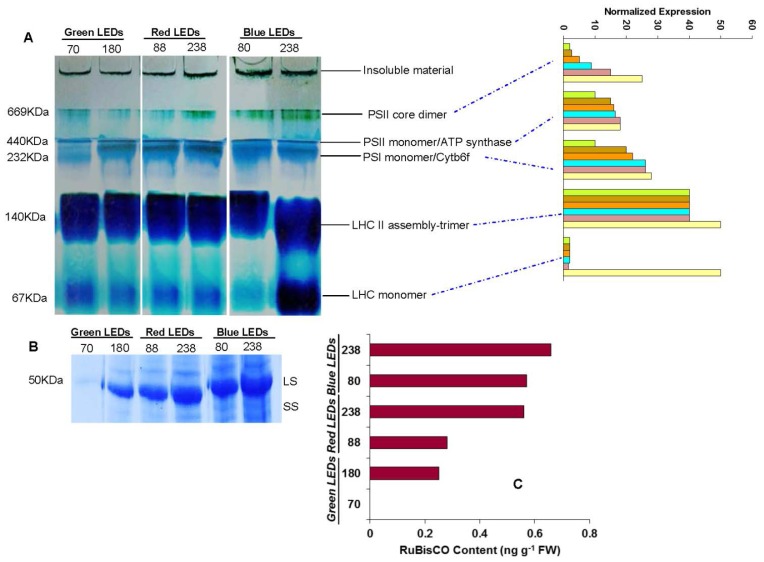
(**A**) Analysis of thylakoid protein complex by BN-Page; (**B**) RuBisCO determination by SDS-PAGE; (**C**) and quantification as affected by green, red and blue LEDs at different light intensities green (70 and 180 μmol m^−1^ s^−1^), red (88 and 238 μmol m^−1^ s^−1^) and blue (80 and 238 μmol m^−1^ s^−1^). Freshly thylakoid membranes from the leaves were solubilized in 1% BDM at a chlorophyll concentration of 1 μg μL^−1^, and the protein sample was separated by 7%–10% gradient BN-PAGE.

First dimensional electrophoresis run under native conditions on BN-PAGE were used to separate intact multiprotein complexes from thylakoids isolated from mature leaves as affected by different light intensities and different LEDs (Figure 4). Gel portions between 1000 and 669 kDa contained PSII PSII-core dimer super complex bands (band 1). The intensity of these bands was observed highest at 238 μmol m^−2^ s^−1^ and lowest at 80 μmol m^−2^ s^−1^ in plants grown under blue LEDs (also see normalized expression). Band 1 at 238 was higher and lower, respectively, at 88 μmol m^−2^ s^−1^ in plants grown under red LEDs. These bands were very faint at 70 μmol m^−2^ s^−1^ in plants grown under green LEDs. In contrast, the intensity of this band was highest in plants grown under blue LEDs than red and green LEDs at different light intensities. The blue band at 440–232 kDa (band 2 and 3) contained the PSII monomer/ATP synthase and PSI monomer/Cytb6f. Reduction of this band was marked in green LEDs at 70 μmol m^−1^ s^−1^ and was observed to be highly expressed at blue LEDs at 238 μmol m^−1^ s^−1^. Analogously strong variation was observed at 140 kDa (band 4), which contained LHCII (light harvesting complex) assembly trimer. This band was expressed in almost all conditions. However, the intensity was marginally higher in plants grown under blue LEDs at 238 μmol m^−1^ s^−1^ and lower in plants grown under red and green LEDs at different light intensities. A LHCII (light harvesting complex) monomer was identified at 67 kDa (band 5) remained unaffected under all light sources and light intensities, except a strong variation was observed at 238 μmol m^−1^ s^−1^ under blue LEDs.

For RuBisCo content quantification was performed after sodium dodecyl polyacrylamide gel electrophoresis (SDS-PAGE) (Figure 4B,C). The intensity of RuBisCO was observed highest at 238 μmol m^−2^ s^−1^ and lowered at 80 μmol m^−2^ s^−1^ in plants grown under blue LEDs. The intensity of RuBisCO was strongly reduced at green LEDs at 180 μmol m^−2^ s^−1^ and absent at 70 μmol m^−2^ s^−1^.

## 3. Discussion

The structure and physiology of plants are particularly regulated by light signals from the environment [4,20], as the primary response of plants during photosynthesis completely depends on light conditions. Plant growth and productivity depends on the light conditions [21] and photosynthetic metabolism is detrimentally affected by light intensity. Plants have developed a sophisticated mechanism to adapt their structure and physiology to the light environment. In this study, we demonstrate that blue LEDs with high light intensity superimpose over red and green LEDs. Plants grown under blue LEDs successfully induced maximum Ψ_W_ (water potential) to −2.33 MPa and fell to a minimum value of −0.233 MPa in leaves of plants grown at green LEDs (Figure 1E). Exposure to green LEDs reduces biomass at low light intensity and a biomass increase was observed under blue LEDs at 238 μmol m^−1^ s^−1^. These results give a clear indication that blue LEDs in combination with high light intensities are more efficient for biomass production in plants. Red and blue light is important for expansion of the leaf and enhancement of biomass [22–24]. Yorio *et al*. [5] reported that there was higher weight accumulation in lettuce grown under red light supplemented with blue light than in lettuce grown under red light alone. However, the shoot dry matter weight of leaf lettuce plants irradiated with blue light decreased compared with that of white light [25]. In the present experiments, blue LEDs in combination with high light intensity was important for growth elongation and biomass accumulation compared to plants grown under low light intensities.

Physiological studies of photosynthesis conducted for many years have considered various light conditions. A combination of red and blue LEDs is an effective source for photosynthesis [16] using different light intensities and wave lengths. Blue LEDs deficiency can result in acclimations of light energy partitioning in PSII and CO_2_ to high irradiance in spinach leaves [7]. Presently, lettuce plants depended on high light intensity (Figure 2) and LEDs for higher rate of photosynthesis. The higher rate of photosynthesis at 238 μmol m^−1^ s^−1^ in plants grown at blue LEDs indicated that lettuce plants displayed pronounced acclimation of photosystems for CO_2_ fixation than plants grown under red and green LEDs. A lower photosynthetic rate in plants grown under red LEDs has been observed in several crops including rice [8] and in wheat [3]. The reduced rate of photosynthesis under low light intensity and red LEDs suggests that vulnerability to a decreased the photosynthetic rate might be associated with changes in multiprotein complexes (PSI and PSII). The lower rate of photosynthesis in red LEDs can also be attributed to low nitrogen content in leaves, due to low chlorophyll and carotenoid content, which was also observed in the present study (data not shown) [26].

The stomata are important channels for the exchange of water and gases with external environmental conditions. Light influences stomata conductivity and proton motive forces [27]. The development of stomata has been related to light intensity [28]. Our results agree with these previous findings and additionally show that blue LEDs are more efficient in stomatal structure and opening and closing of stomata (Figure 3). The number of stomata increased more in plants grown under blue LEDs at 238 μmol m^−1^ s^−1^ compared to plants grown under low light intensities and other LEDs. The closure and reduced number of stomata might be due to defoliation of leaves under low light intensity during growth of lettuce. Indeed, high temperatures under different light intensity conditions might induce palisade and increased sponge parenchyma cell length and thickness [29]. The closure of stomata with reduced normalized expression and number might be also the reason for reduction of transpiration rate and stomatal conductance in lettuce which were grown under green LEDs more so than those grown under blue LEDs.

The thylakoid membranes are the sub-compartments in which the primary reactions of photosynthesis occur. About 100 proteins are involved in these reactions; they are organized in four major multisubunit protein complexes: PSI, PSII, ATP synthase complex and cytochrome b6/f (cyt b6/f) complex [30]. Proteomics of the thylakoid membrane are an excellent approach to establish the number and identity of the proteins localized to this sub-compartment in pigment–multiprotein complexes, and to study the impact of light intensity and light source on them for increased photosynthetic metabolism and other physiological process. Several diverse photosynthetic factors have been observed at different light intensities with inhibition of photosynthetic factors associated with carbohydrate metabolism in leaves [31]. However, to date there is no information on the expression of thylakoid proteins under different intensities of light and light sources. Our results show that the induction in the expression of PSII-core dimer under blue LEDs at 238 μmol m^−1^ s^−1^ (Figure 4A). The reduction of these multiprotein complexes at red and green LEDs might limit mineral nutrient clusters which are associated with the chloroplast membrane [32]. In addition to this, leaves exposed to green LEDs might reject light due to chlorosis that occurs due to proteolytic loss of photosystems and the cytb*6*/f complex [33] and the light-harvesting chlorophylls and carotenoids. The inhibition of PSI and PSII under red and green LEDs with low light intensity suggests the involvement of an unidentified problem related to transport of substances in plants are due to reduced amounts of core antenna Chl-protein complexes [34]. The involvement of blue LEDs at high light intensity leads to maintenance of PSI and PSII core complexes. In some reports, it has been postulated that the intensity of blue light for activation of PSII core protein content in *Arabidopsis* acting via cryptochromes, along with non-blue specific activation signals

Our data clearly show that RuBisCO was expressed at 238 μmol m^−1^ s^−1^ whereas it was absent in plants grown under green LED light sources (Figure 4B,C) which were positively paralleled with other multiprotein complexes. The enhancement of RuBisCO under high intensity of blue LED might be associated with an increase in the amount of N content accompanied by induction of chlorophyll content or it might be also due to wider and thinner expansion of palisade and sponge parenchyma. The induction of RuBisCO in plants grown under blue LED light might be also due the expansion of palisade and sponge parenchyma. Reduction of thylakoid protein complexes and photosynthetic parameters under green and red LEDs at low light intensity indicate a close dependence of the photosynthetic metabolism on the source of light and its intensity. The proteins of chloroplast sub-compartments under blue LEDs at high light intensity optimize photosynthesis and provide an advantage for higher growth and development of plants than those grown under red and green LEDs at low light intensities.

## 4. Material and Methods

### 4.1. LEDs of Different Light Intensities

All combined LEDs had different spectra of green, red, and blue light. Light treatments for young lettuce plants were 70 and 180 μmol m^−2^ s^−1^ for green, 88 and 238 μmol m^−2^ s^−1^ for red, and 80 and 238 μmol m^−2^ s^−1^ for blue. Photon flux density (PPFD) was measured using a LI-250 quantum sensor (LI-COR, Lincoln, NE, USA) and was separately controlled by adjusting both electric currents and number of light bulbs for the LEDs. The wavelengths of different light intensities are shown in Table 2. All treatments were done in a culture room, employing separate plots for the different light intensities. The room was ventilated to maintain the CO_2_ level the same as that of the outside atmosphere. The relative humidity was maintained at 70% ± 10% with a 16 h photoperiod and a temperature of 25 °C during the light period and 18 °C during the dark period.

### 4.2. Plant Material and Growth Conditions

Red-wrinkled lettuce seeds (“Hongyeom”, Sakata Korea Seed, Seoul, Korea) of *Lactuca sativa* L. were sown in 240 cells of Rockwool tray with electrical conductivity of 1.5 dS m^−1^ and were germinated at 25 °C under florescent light. The seedling with 5 true leaves seven days after sowing was transplanted on the growing system of deep flow technique (DFT) using commercially solid nutrient [35] (Global Coseal, Limited, Seoul, Korea) diluted in tap water with EC 1.53 dS m^−1^ with pH 5.9. The plants were randomized into eight groups and were placed under 8 light treatments for 15 days. All measurements were carried out using the fully expanded mature leaf of the plant.

### 4.3. Growth Measurements and Ψ_W_ Potential

Plants were uprooted carefully from the hydroponic solution and blot-dried with soft lint free paper. Each plant was separated into roots, stem, and leaves using a sharp scalpel and forceps in moist paper sheets. The biomass of the leaf, root, and stem fractions was determined. For dry biomass determination, plant material was dried at 65 °C for 2 days and weighed on an electronic weighing balance.

After fresh and dry weight of samples following formula was used to calculate leaf water potential.

Relative water content (RWC) %=(FW-DW)/(TM-DW)×100

where FW indicates fresh weight, DW indicates dry weight and TM indicates turgid weight.

### 4.4. Measurement of Photosynthetic Activity

Photosynthetic rate, transpiration rate, and stomatal conductance were measured using a LI-6400XT portable photosynthesis measurement system (LI-COR, John Morris Scientific, Sydney, Australia). Gas exchange was measured on the fully expanded mature leaves at 20 °C inside the clutch with CO_2_ concentration maintained at 600 μmol mol^−1^. Chlorophyll fluorescence (Fv/Fm) was measured by using a PAM 2000 chlorophyll fluorescence meter (Heinz Walz GmbH, Effeltrich, Germany). The leaves were adapted to dark conditions for 30 min before measurement. The maximum fluorescence (Fm) and minimum fluorescence (F_0_) were determined by applying a saturating light pulse (20 kHz) of 1100 μmol·m^−2^·s^−1^ PPF for 3 μs. The maximum PS II quantum yield (Fv/Fm) was calculated as Fv/Fm = (Fm − F_0_)/Fm.

### 4.5. Observation of Stomata

For stomatal observation, thin layers of leaf tissues were carefully cut and were laid on a glass slide, covered with a cover slip by adding a few drops of glycerine, and were observed using a DM4000 light microscope (Leica, Wetzlar, Germany) at 10× and 40× magnification. The number of stomata was observed by counting the number in the present leaf area. The stomatal density was calculated by dividing the number of stomata counted by 10 times the area of 1 grid square.

### 4.6. Multiprotein Complex Proteins

Blue native-polyacrylamide gel electrophoresis (BN-PAGE) of integral thylakoid proteins was performed as previously described [36]. Five grams of fresh leaf tissues were homogenized in liquid nitrogen and thylakoid membranes were extracted using an extraction buffer (pH 7.8) containing 20 mM Tricine-NaOH, 70 mM sucrose, and 5 mM MgCl_2_ and were filtered through miracloth/cheesecloth before centrifugation at 4500× *g* for 10 min. The thylakoid pellet was resuspended in the same buffer (pH 7.8) and centrifuged again. The resulting pellet containing thylakoid membranes was washed and extracted with each proper buffer. An equal volume of resuspension buffer containing 2% (*w*/*v*) *n*-dodecyl-β-d maltoside (Sigma-Aldrich, St. Louis, MO, USA) was added under continuous mixing and the solubilization of membrane-protein complexes was allowed to occur for 3 min on ice. Insoluble material was removed by centrifugation at 18,000× *g* for 15 min. The supernatant was mixed with 0.1 volume of 5% *w*/*v* Serva blue G, 100 mM Bis Tris-HCl (pH 7.0), 30% *w*/*v* sucrose, and 500 mM €-amino-*n*-caproic acid and loaded onto a 0.75-mm-thick 5%–12.5% *w*/*v* acrylamide gradient gel (180 × 160 mm). Electrophoresis was performed at 4 °C by increasing the voltage from 100 to 200 V overnight.

### 4.7. RuBisCO Determination by SDS-PAGE

Leaf tissues were homogenized at 4 °C in 100 mM Tris buffer (pH 7.5) containing 5 mM of DTT, 2 mM iodoacetate and 5% (*v*/*v*) glycerol at a leaf; buffer ratio of 1:5–10 (g:mL). For this extraction, a buffer without sodium or potassium ion was recommended for SDS-PAGE analysis because those cations reduce the solubility of DS (dodecyl sulfate). Before centrifugation, a TritonX100 (25%, *v*/*v*) was added to a portion of leaf homogenate to make a final concentration of 0.1% (*v*/*v*). An addition of TritonX100 was effective for the extraction of RuBisCO bound to the membrane fraction. The homogenates were centrifuged at 5000× *g* for 3 min at 4 °C. A lithium DS solution (25% *w*/*v*) and 2-mercaptoethanol were added to the supernatant fluid to a final concentration of 1.0% (*w*/*v*) and 1% (*v*/*v*), respectively. This preparation was immediately treated at 100 °C for 1 min, and was then stored at −30 °C until the analysis of SDS-PAGE. The samples containing 2–10 μg RuBisCO were loaded onto 12% polyacrylamide gel. After electrophoresis, the gels were stained in 0.25% (*w*/*v*) CBB-R. The stained bands corresponding to larger and smaller subunits of RuBisCO were cut out of the gels with a razor blade and were eluted in 1–2.5 mL of formamide in a stoppered amber test tube at 50 °C for 5 h with shaking. The absorbance of the resultant solution was read at 595 nm with a spectrophotometer. RuBisCO content was determined by using the standard curve calculated from the absorbance of a known amount of purified RuBisCO.

### 4.8. Statistical Analysis

A completely randomized design was used with five replicates for six treatments. An individual Student’s *t* test and Tukey’s studentized range test was employed to compare the means of separate replicates by using SAS version 9.1 (SAS Institute, Cary, NC, USA).

## 5. Conclusions

Finally, we conclude that blue LEDs at high light intensity promote plant growth by controlling the integrity of chloroplast proteins that elevates photosynthetic performance in the natural environment. Further analysis in multiprotein complex proteins followed by the second dimension along with genomic data will provide important information for development of plants with better with-standing potential under different light intensities and LED conditions.

## Figures and Tables

**Table 1.** Stomatal densities at respective different LEDs with different light intensities.

Light intensity (μmol m^−2^ s^−1^)	Stomatal density (Mean ± SE, mm^2^)		

	Green LEDs	Red LEDs	Blue LEDs
70	0.7 ± 0.11		
180	1.0 ± 0.11		
88		1.4 ± 0.11	
238		1.85 ± 0.11	
80			1.7 ± 0.11
238			2.3 ± 0.11

**Table 2.** Major light wavelengths of different light intensities.

Light sources	Light intensity (μmol m^−2^ s^−1^)	Peak wave length λp (nm)
Green	70	522
Green	180	522
Red	88	639
Red	238	639
Blue	80	470
Blue	238	470
